# Cell-specific housekeeping role of lncRNAs in COVID-19-infected and recovered patients

**DOI:** 10.1093/nargab/lqae023

**Published:** 2024-02-29

**Authors:** Partha Chattopadhyay, Priyanka Mehta, Jyoti Soni, Kishore Tardalkar, Meghnad Joshi, Rajesh Pandey

**Affiliations:** Division of Immunology and Infectious Disease Biology, INtegrative GENomics of HOst-PathogEn (INGEN-HOPE) laboratory, CSIR-Institute of Genomics and Integrative Biology (CSIR-IGIB), Mall Road, Delhi-110007, India; Academy of Scientific and Innovative Research (AcSIR), Ghaziabad-201002, India; Division of Immunology and Infectious Disease Biology, INtegrative GENomics of HOst-PathogEn (INGEN-HOPE) laboratory, CSIR-Institute of Genomics and Integrative Biology (CSIR-IGIB), Mall Road, Delhi-110007, India; Academy of Scientific and Innovative Research (AcSIR), Ghaziabad-201002, India; Division of Immunology and Infectious Disease Biology, INtegrative GENomics of HOst-PathogEn (INGEN-HOPE) laboratory, CSIR-Institute of Genomics and Integrative Biology (CSIR-IGIB), Mall Road, Delhi-110007, India; Academy of Scientific and Innovative Research (AcSIR), Ghaziabad-201002, India; Department of Stem Cells & Regenerative Medicine, D.Y. Patil Education Society, Kadamwadi, Kolhapur-416003,Maharashtra, India; Department of Stem Cells & Regenerative Medicine, D.Y. Patil Education Society, Kadamwadi, Kolhapur-416003,Maharashtra, India; Division of Immunology and Infectious Disease Biology, INtegrative GENomics of HOst-PathogEn (INGEN-HOPE) laboratory, CSIR-Institute of Genomics and Integrative Biology (CSIR-IGIB), Mall Road, Delhi-110007, India; Academy of Scientific and Innovative Research (AcSIR), Ghaziabad-201002, India

## Abstract

A plethora of studies have demonstrated the roles of lncRNAs in modulating disease severity and outcomes during infection. However, the spatio-temporal expression of these lncRNAs is poorly understood. In this study, we used single-cell RNA-seq to understand the spatio-temporal expression dynamics of lncRNAs across healthy, SARS-CoV-2-infected, and recovered individuals and their functional role in modulating the disease and recovery. We identified 203 differentially expressed lncRNAs, including cell type-specific ones like *MALAT1*, *NEAT1*, *ZFAS1, SNHG7, SNHG8, and SNHG25* modulating immune function in classical monocyte, NK T, proliferating NK, plasmablast, naive, and activated B/T cells. Interestingly, we found invariant lncRNAs (no significant change in expression across conditions) regulating essential housekeeping functions (for example, *HOTAIR, NRAV, SNHG27, SNHG28, and UCA1*) in infected and recovered individuals. Despite similar repeat element abundance, variant lncRNAs displayed higher Alu content, suggesting increased interactions with proximal and distal genes, crucial for immune response modulation. The comparable repeat abundance but distinct expression levels of variant and invariant lncRNAs highlight the significance of investigating the regulatory mechanisms of invariant lncRNAs. Overall, this study offers new insights into the spatio-temporal expression patterns and functional roles of lncRNAs in SARS-CoV-2-infected and recovered individuals while highlighting the importance of invariant lncRNAs in the disease context.

## Graphical Abstract

**Graphical Abstract:**
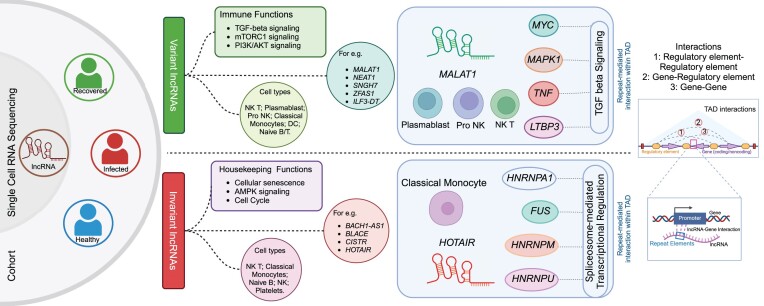

## Introduction

During host-pathogen interactions, several protein-coding genes and signaling molecules are known to be activated and play functional roles in disease severity and clinical outcome. With advances in genomics, long non-coding RNAs (lncRNAs) have also emerged as another part of the host transcriptome that is crucial during infection. A flurry of findings have highlighted the role of lncRNAs in the regulation, progression, and outcome of infectious diseases, not only during the antiviral response (1) but also in ways that can be beneficial to the pathogen (2). For example, lncRNA *EGOT* (Eosinophil Granule Ontogeny Transcript) antagonizes the antiviral response and promotes the proliferation of the hepatitis-C virus (HCV) (3). LncRNAs also play a role in viral replication by controlling viral gene transcription, translocation, and protein function. *NEAT1* (Nuclear Enriched Abundant Transcript 1), an abundant lncRNA, is known to have varied effects on viruses. It inhibits human immunodeficiency virus (HIV) replication via stimulating interferon (IFN) signaling or by increasing the translocation of *cis-*acting instability elements (INS)-containing viral RNAs, whereas it enhances herpes simplex virus (HSV) replication by enabling STAT3 (signal transducer and activator of transcription 3) binding to viral gene promoters (4–9). Thus, as one of the key regulators of the innate immune response, lncRNA expression can have both positive and negative effects on the host response to pathogens.

Although lncRNAs affect gene expression by influencing transcriptional and post-transcriptional modifications, their expression varies greatly depending on the cell type and tissue specificity (10). A study by Cabili *et al.* found 78% of lncRNAs to be tissue-specific across the 24 cell types and tissues in comparison to only 19% of protein-coding genes (11). LncRNAs, in general, have shorter transcript lengths than protein-coding genes as they tend to carry a lower number of exons (12). Bulk expression studies have shown that lncRNAs are expressed at lower levels than mRNAs. It is unknown whether this directional selectivity of lncRNA expression is due to universally decreased expression of lncRNAs or higher expression in specific cells in comparison to mRNAs, making it vital to investigate their cell- and tissue-specific activities (5–7,13,14). Therefore, investigating cellular diversity and heterogeneity is crucial during infection, and single-cell techniques can reveal subpopulation variances that might influence outcomes and individual differences.

Single-cell genomics has revealed granular insights on host-pathogen interactions. Investigations using blood-derived peripheral blood mononuclear cells (PBMCs) from COVID-19 patients show that interferon (IFN)-stimulated genes (ISGs) are upregulated in cell types such as B cells/plasma cells, T cells, NK cells, monocytes, neutrophils, and others, demonstrating the antiviral activities of these cells (15–18). ISG expression was found to be more prevalent in patients with moderate disease severity than in those with severe disease, indicating a reduction in immunity that leads to severity. Similarly, downregulation of human leukocyte antigen (HLA)-II genes in infected patients’ B and myeloid cells relative to healthy individuals reveals a dysregulated immunological interaction (5–7,19–21). Furthermore, genes encoding inflammatory cytokines, CCL2/CCL3/CCL4, and proinflammatory molecules, S100A8/A9, were found to be elevated in various cells, indicating hyperinflammation during infection and subsequently contributing to the cytokine storm characteristic of severe COVID-19 (5–7,16,22,23). These findings highlight the heterogeneous dynamics of host response in diverse immune cell types along with the trajectory of immune response from infection to recovery (24). However, a common focus of all these studies has been the protein-coding genes and surface markers involved in the immune response. While assessing the evidence on lncRNAs role in influencing immune response during infection, it is imperative to focus on understanding the functional duet between the lncRNAs and the mRNAs within diverse cell types during infection.

While the above insights help address how and when the host responds to the infection, they do not address why similar groups of people with the same/similar pathogen challenges have different disease outcomes. As seen during the COVID-19 pandemic, clinical severities varied from mild, moderate, and severe to even fatality, while the underlying pathogen was isogeneic. Few studies, including our previous studies, have highlighted genes and lncRNAs underlying differential disease severity and outcomes in COVID-19 (25–28). However, host responses post-SARS-CoV-2 infection, i.e., in the recovered individuals, are yet to be understood in depth, especially when limited evidence (including from our group) reports heightened inflammatory responses, loss of B cell maturation, and T cell exhaustion in the recovered individuals (24).

**Figure 1.**
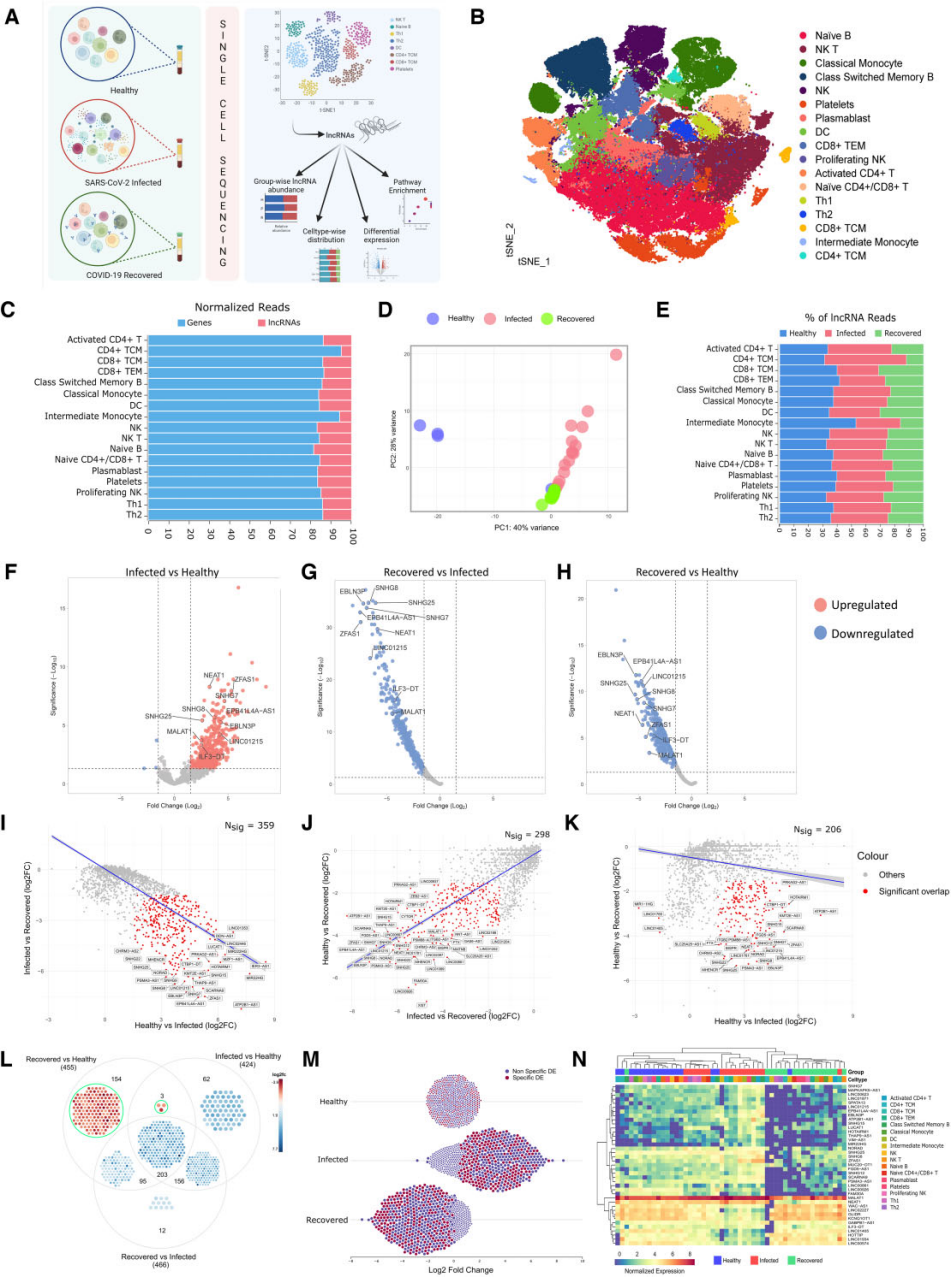
Differential expression pattern of lncRNAs and cell type dynamics across the healthy, infected, and recovered individuals captured by the scRNA-seq. (**A**) Graphical representation of a single-cell study design to investigate the lncRNA expression patterns within the immune cells of healthy, SARS-CoV-2-infected, and recovered individuals. (**B**) tSNE plot representing the 17 clusters of cells identified across healthy, infected, and recovered. (**C**) Proportion of lncRNA reads (in red) captured in the whole transcriptome data with respect to mRNA reads (in blue) in the different cell types. (**D**) PCA plot of the sample level clustering showing a distinct difference with respect to the lncRNA expression between the three groups. (**E**) The stacked bar plot represents the cell type-wise distribution of lncRNA reads between healthy (blue), infected (red), and recovered (green) individuals. (F–H) The volcano plot represents the significantly differential expression of lncRNAs between (**F**) infected vs. healthy, (**G**) recovered vs. infected, and (**H**) recovered vs. healthy groups at *P* adjusted value ≤0.05 and log_2_fc |1.5|, where downregulated lncRNAs are represented in red and upregulated lncRNAs in blue. (I-K) lncRNAs are differentially expressed between (**I**) healthy vs infected and infected vs recovered (**J**) healthy versus recovered and infected vs. recovered, and (**K**) healthy vs. recovered and healthy versus infected comparison. The lncRNAs significantly differentially expressed in both comparison groups are highlighted in red. The number of overlaps of significant DE genes between the two comparison groups is marked as ‘Nsig’. (**L**) The Venn diagram depicts the lncRNAs that overlap between the three comparison groups. The red circles show upregulated DEGs, and the green circles represent the downregulated genes in the respective groups. (**M**) The dot plot depicts the log2 fold change of the DE lncRNAs between healthy, infected, and recovered, where red dots represent the 203 common lncRNAs between the three groups. (**N**) The heatmap depicts the normalized expression of the top lncRNAs that differed in all three groups, along with the cell type in which they are expressed.

In this study, we investigated the spatio-temporal expression of lncRNAs at single cell resolution in healthy, active SARS-CoV-2 infection and COVID-19-recovered individuals. We performed lncRNA-specific differential expression analysis, both at the pseudo-bulk and cell-level resolutions, to identify the dysregulated set of lncRNAs that modulate the immune response in COVID-19-positive and, importantly, recovered individuals. The experimental workflow is depicted in Figure 1A and Supplementary Figure S1. Importantly, we identified a set of lncRNAs that are expressed during the pathogen challenge, but their expression did not change from healthy to infected and recovered individuals (henceforth denoted as invariant lncRNAs). We demonstrated that the invariant lncRNAs are responsible for maintaining essential housekeeping functions during and after SARS-CoV-2 infection and contribute to host response vis-à-vis SARS-CoV-2 infection. We also identified class-switched memory B cells, naive B cells, classical monocytes, NK T cells, NK cells, and platelets as key cell types responsible for this invariant lncRNA-mediated regulation of housekeeping functions. Interestingly, we observed a higher abundance of repeats (Alu elements) within the variant lncRNAs compared to the invariant, both at the cDNA and the promoter region. Notably, the variant lncRNAs had higher interactions with both the proximal and distally located protein-coding genes than the invariant lncRNAs. It suggests Alu-mediated increased interaction between the variant lncRNAs and adjacent genes is required for modulation of the immune response, while fewer interactions are required for the invariant lncRNAs to modulate the housekeeping functions in the SARS-CoV-2-infected and recovered individuals.

## Materials and methods

### Ethics statement

The studies involving human participants were reviewed and approved by CSIR-IGIB’s Human Ethics Committee Clearance (Ref. No.: CSIR-IGIB/IHEC/2020-21/01). The patients and participants provided their written informed consent prior to their participation in this study. The patients and participants provided their written informed consent to participate in this study. The PBMCs were isolated from freshly collected blood at a tertiary care center: Dr. D.Y. Patil Medical College, Hospital and Research Institute, Kolhapur, Maharashtra, India. A total of 33 age- and gender-matched volunteers, including 4 healthy, 16 qRT-PCR-positive COVID-19 patients, and 13 recovered individuals (within 4 weeks post-first qRT-PCR-negative), were recruited in this study. The study was approved by the CSIR-IGIB Human Ethics Committee Clearance (Ref No: CSIR-IGIB/IHEC/2020–21/01). The studies were conducted in accordance with the Declaration of Helsinki as well as local legislation and institutional requirements. The participants provided their written informed consent to participate in this study.

### Sample collection, processing and library preparation

The PBMCs were isolated using a BD Vacutainer® CPTTM Cell Preparation Tube as per the manufacturer's recommendation. A single-cell Whole Transcriptome Analysis (WTA) library was prepared using the BD RhapsodyTM Whole Transcriptome Analysis (WTA) Amplification Kit and the BDTM Single-Cell Multiplexing Kit-Human as described earlier (24). Initially, at least three samples with an equal number of cells (2 lakhs per sample) were pooled, and at the end, a total of 30 000 cells were loaded in each cartridge on the BD Rhapsody Express single cell analysis system for single cell capture, followed by cDNA synthesis as per the manufacturer's guidelines (Doc ID: 210967 Rev. 1.0). The libraries were sequenced on the NovaSeq 6000 platform using the S2 reagent kit at 30 000 reads/cell and 101 bp paired end sequencing mode.

### Single-cell RNA-seq (scRNA-seq) data processing, clustering and cell-type annotation

Raw sequencing reads were processed by BD RhapsodyTM WTA Analysis Pipeline, v1.0, Revision 4. The count matrix with recursive substitution error correction was used for downstream analysis in the Seurat v4.3 R package (29). A total of 163,197 cells were detected initially. We then removed the low-quality cells, followed by batch effect removal and normalization using Seurat SCTransform V2 (29,30), and cells were clustered at a resolution of 0.4. Unsupervised clustering was performed by the KNN-based Louvain algorithm, and the clusters were identified manually using CellMarkerDB and PanglaoDB, as well as automated annotation by Azimuth and scPred (31–33).

### Differential expression of mRNAs and lncRNAs

The total reads were segregated into coding and non-coding reads, and the non-coding reads were extracted for each cell type vis-à-vis groups. Principal component analysis was performed within Seurat, and pseudobulk differential expression analysis was performed for both lncRNA and mRNA genes using the DESeq2 R package (34). The Wald test was applied for the identification of differentially expressed lncRNAs and mRNAs, and genes with a log fold change of ≥±1.5 and a significance value (*P* adjusted) ≤0.05 were considered to be significantly differentially expressed. The differentially expressed lncRNAs and mRNAs were plotted using ggplot2, Rawgraph (https://github.com/rawgraphs), and the pheatmap package (https://github.com/raivokolde/pheatmap).

### Gene ontology and pathway enrichment analysis

LncRNA-mRNA interactions were curated from NPInter v4.0, a database for functional interactions between non-coding RNAs and other biomolecules (35). Protein-coding genes with experimentally validated interactions with lncRNAs were used for GO and pathway enrichment analysis using gProfiler and Enrichr (5–7,36,37) A significance threshold of *P*-adjusted value ≤0.05 was set, and the significant pathways were visualized using ggplot2. Further, to investigate whether the pathways identified through gene ontology and pathway enrichment analysis are actually perturbed or not, we performed gene set enrichment analysis (GSEA) using the genes that are differentially expressed and have reported interaction with variant/invariant lncRNAs. The genes were ranked according to their expression, and the enrichment analysis was performed using ShinyGO v 0.77, with total expressed genes as background (38).

### Cell type-specific expression module

To group the lncRNA expression into multiple modules and interpret the function of each module, we used CEMitool and applied a Pearson correlation with a significance level of ≤0.05 (39). The cell type-wise count matrix and the cell type annotation were used for the analysis. The invariant lncRNAs were clustered into two modules, and the genes interacting with lncRNAs present in each module were taken forward for pathway enrichment using Enrichr. To test the null hypothesis, cell type-specific expression data was obtained from the Blueprint Epigenome Project (40) and pathway enrichment analysis was performed using Enrichr as described earlier.

### LncRNA biotype and repeat element distribution

The lncRNAs were categorized with respect to their biotype and origins, as described in the LncRBase v2.0 database (41). The RepeatMasker web tool was used to annotate the repeat elements within the body as well as the promoter region of the lncRNAs (42). RepeatMasker uses the Dfam 3.0 database and the rmblast algorithm to identify the repeats (43). The abundance of repeat elements was normalized against the total bases of the input sequence and is represented as the number of repeat bases per kbp. The chi square test was used to test the significance of the repeat abundance between two groups using the graphpad prism (www.graphpad.com).

### Proximal and distal gene regulation

We extracted the genes present in proximity of the lncRNAs (±5 kb region) from the UCSC Genome Browser and matched the genes with the experimentally validated interacting protein-coding genes obtained from the NPInter database for those lncRNAs. We then performed pathway enrichment for those adjacent genes to identify the pathways that are possibly regulated by these lncRNAs.

In order to understand the distal gene regulation, we identified the topologically associated domains (TAD) in which the lncRNAs are present. We used the TADKB database to get the coordinates of the TADs, experimentally obtained from K562 cell lines at 10 kb resolution (44). We then identified the other genes present within the TAD and predicted the interaction between the lncRNA and other genes present within the same TAD using the Triplex Domain Finder tool (45). The number of interactions between lncRNA and mRNAs is plotted using the ggplot2 R package. Mann–Whitney *A U* test was applied to check the significance of the difference between the number of interactions.

## Results

### Differential cell type-specific enrichment of lncRNAs in SARS-CoV-2-infected patients

To understand the lncRNA-mediated host response vis-à-vis cell types in the COVID-19 patients and recovered individuals, we used the BD Rhapsody express single cell analysis system to perform the single-cell RNA-seq of four healthy, 16 SARS-CoV-2-infected, and 13 recovered individuals (Figure 1A, Supplementary Table S1). We identified a total of 163 197 cells initially, and after filtering/removal of low-quality cells, 124 726 cells were used for downstream analysis, including normalization, clustering, cell type identification, and differential expression analysis. Within our cohort, we identified 17 different cell types. (Figure 1B), within which a consequential fraction (∼15–20%) of the total reads were mapped to the noncoding genes (Figure 1C). Using the lncRNA expression data, we performed principal component analysis and found a strong contrast between the healthy and the infected and recovered groups (Figure 1D). Nevertheless, there was no difference in cell type-specific lncRNA expression across the healthy, infected, and recovered individuals (Figure 1E). Therefore, to identify the differentially expressed lncRNAs across the three groups (healthy, infected, and recovered), we performed pseudo-bulk differential expression using DESeq2 between the three comparison groups: infected vs. healthy, recovered vs. infected, and recovered vs. healthy (Figure 1F–H).

We identified 424 differentially expressed (DE) lncRNAs between the infected and healthy individuals (421 up and 3 downregulated in the infected), 466 DE lncRNAs between the recovered and infected (all upregulated in the infected), and 455 DE lncRNAs in the recovered vs. healthy (all downregulated in the recovered) (Figure 1L, Supplementary Table S2A-S2C). Of the 466 upregulated lncRNAs in the infected (versus recovered), 359 lncRNAs were also upregulated in the infected compared to the healthy individuals, and 298 lncRNAs were downregulated in the recovered individuals compared to the healthy. For example, lncRNAs like *SNHG8*, *SNHG9*, *SNHG25, NEAT1, NORAD, LUCAT1, CYTOR, BISPR*, and *MALAT1* were upregulated in the infected (vs. recovered) but downregulated in the recovered individuals compared to the healthy. Between the healthy vs. infected and healthy vs. recovered groups, 206 lncRNAs were downregulated in the recovered group and upregulated in the infected group (Figure 1I-K). For example, *SNHG8, ZFAS1, NEAT1, LINC01215, HOTAIRM1*, and *NORAD* were significantly upregulated in the infected individuals but downregulated in the recovered compared to the healthy. Cumulating all the differentially expressed lncRNAs across the three groups, we identified a total of 203 lncRNAs to be upregulated in the infected compared to the healthy and downregulated in the recovered compared to both the infected and healthy individuals (Figure 1L,M). For example, expressions of *SNHG7, SNHG8, SNHG9, ZFAS1, BISPR, MALAT1, LUCAT1, NORAD, NIFK-AS1*, and *CYTOR* were upregulated in the infected (vs. healthy) but downregulated in the recovered individuals compared to both infected and healthy. The expression of the top 10 differentially expressed lncRNAs (the top 10 by log fold change, with more than one lncRNA having the same fold change) was plotted across the cell types vis-à-vis three groups, and we observed specific lncRNAs to be expressed in the specific cell types while other lncRNAs did not show change in expression within any specific cell types; however, they were different across the three groups (Figure 1N). This highlights that certain lncRNAs are enriched in specific cell types and study groups, which may have a functional role in modulating the COVID-19 disease trajectory from infected to recovered patients as compared to the healthy.

### Modulation of immune response by the cell type-specific expression of lncRNAs

**Figure 2.**
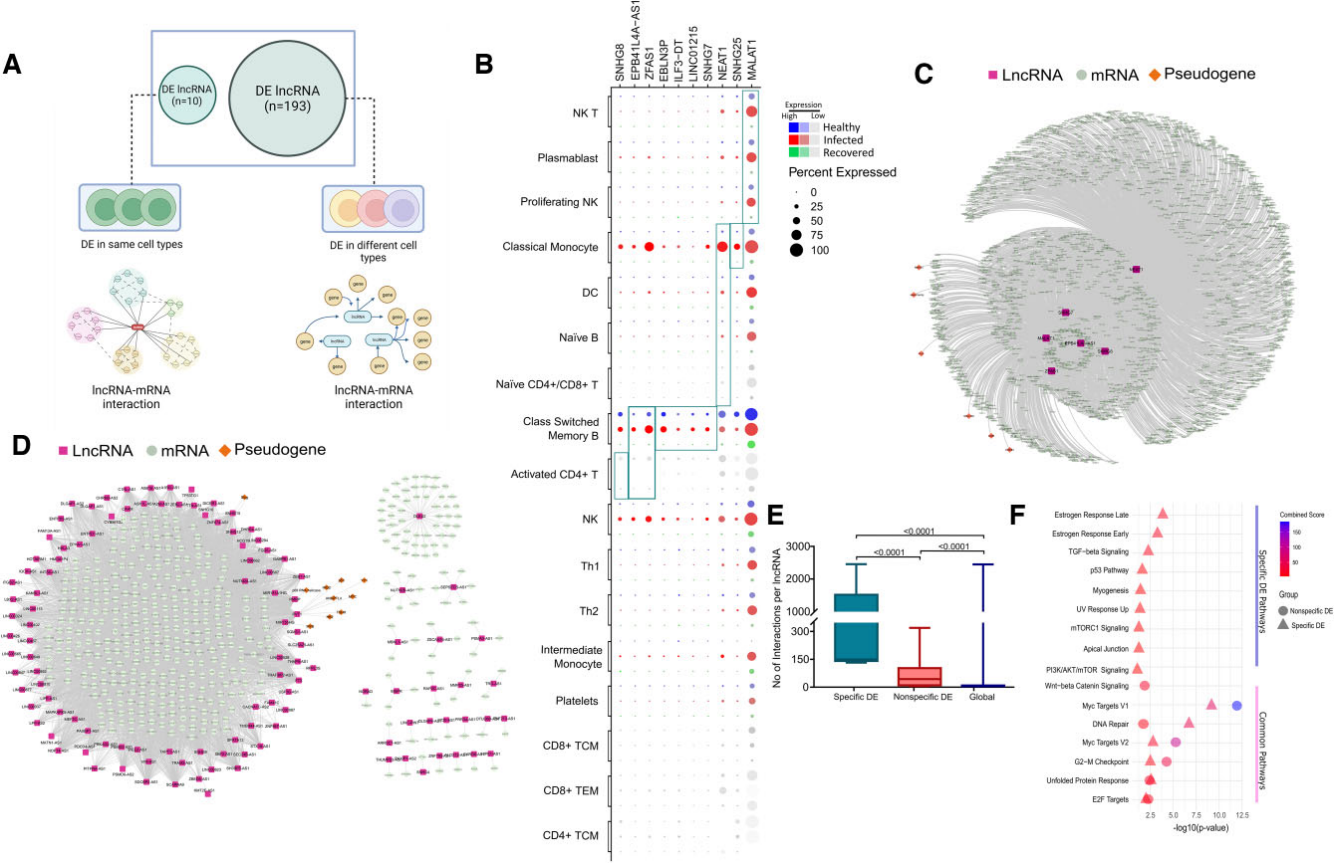
Cell type-specific expression and function of differentially expressed lncRNAs. (**A**) Graphical representation showing the investigation of cell type-specific expression patterns of DE lncRNAs and the lncRNA-mRNA interaction network of cell type-specific (10 DE within the same cell types) and nonspecific DE lncRNAs (19 DE within different cell types). (**B**) Dot plot of the differentially expressed lncRNAs within the same cell type in the three groups (healthy, infectious, and recovered). Cell types in which the lncRNAs were significantly DE are highlighted in a rectangular box. (C, D) The lncRNA-mRNA interaction network for (**C**) 10 lncRNAs expressed differently within the same cell types, and (**D**) 193 lncRNAs expressed differently in the various cell types. Nodes for lncRNAs are depicted as pink squares, whereas nodes for mRNAs are represented as green circles. Orange diamonds reflect regulatory connections between the lncRNA and the mRNA. (**E**) Number of interactions per lncRNA for specific DE and nonspecific DE compared against the global interaction rate. (**F**) The 203 (10 + 193) DE lncRNAs were analyzed for pathway enrichment. Specific DE pathways reflect enrichment owing to the 10 lncRNAs unique to the same cell type, whereas ‘Common Pathways’ represent pathways enriched due to all the 203 DE lncRNAs.

Next, we explored the cell types, specific to differentially expressed 203 lncRNAs, to understand the cell-specific changes in lncRNA expression across healthy, infected, and recovered individuals (Figure 2A, Supplementary Table S3A). We found 10 lncRNAs (*MALAT1, SNHG25, NEAT1, SNHG7, LINC01215, ILF3-DT, EBLN3P, ZFAS1, EPB41L4A-AS1*, and *SNHG8*) to be differentially expressed across the same cell types (Figure 2B). For instance, *MALAT1* was found to be upregulated in the infected and downregulated in the recovered NK T, plasmablast, and proliferating NK cells. *SNHG25* was upregulated in the classical monocytes of the infected. *MALAT1* positively regulates the inflammatory response and negatively regulates the immune response, suggesting a contribution to the inflammatory response by the NK T, pro-NK, and plasmablast in the infected individuals (28)*. NEAT1* was upregulated in classical monocytes, dendritic cells, naive B cells, and naive CD4+/CD8+ T cells. *SNHG7, LINC01215, ILF3-DT*, and *EBLN3P* were upregulated in the infected class-switched memory B cells. *ZFAS1* and *EPB41L4A-AS* were upregulated in the switched memory B and activated CD4+ T cells, while *SNHG8* was upregulated in the activated CD4+ T cells of the infected individuals. *SNHG7, SNHG8*, and *SNHG25*, like *MALAT1* and *NEAT1*, are positive regulators of inflammation and negative regulators of immunological response, implying a function in moderating the inflammatory response within the COVID-19 patients’ classical monocytes, DC, naive B/T, activated CD4 + T, and class switched memory B cells (28,46,47). This cell type-specific enrichment also suggests that these lncRNAs serve specialized/specific functions within these cells during infection, necessitating more investigation into their biological importance. Some of these lncRNAs have also been reported to be involved in other diseases as well, details of which are mentioned in Supplementary Table S3B.

To further understand the various biological functions of these 10-cell type-specific DE lncRNAs and the remaining nonspecific DE lncRNAs (193 lncRNAs), we first retrieved the genes interacting with the lncRNAs using NPInter V4.0 and constructed a lncRNA-mRNA interaction network using experimentally validated interaction data (Supplementary Table S4A). When compared to the nonspecific DE lncRNAs, cell type-specific DE lncRNAs demonstrated a greater degree of interaction (3.37 times higher than nonspecific DE and 37 times higher than global median interactions per lncRNA), or more average interactions per lncRNA (Figure 2C–E). We then performed the pathway enrichment analysis of the genes interacting with both lncRNA categories and observed that the cell type-specific DE lncRNAs are mainly responsible for immune response-related pathways, while the nonspecific DE lncRNAs are involved in diverse biological pathways related to DNA repair, cell cycle, and cell growth regulation (Figure 2F, Supplementary Table S5A). We postulate that the greater level of interaction required by cell type-specific DE lncRNAs is necessary to mediate specific biological activities, in this case, the immune response to a SARS-CoV-2 infection, as compared to the non-specific DE lncRNAs.

### Cell type-specific housekeeping function modulation by the invariant lncRNAs

**Figure 3.**
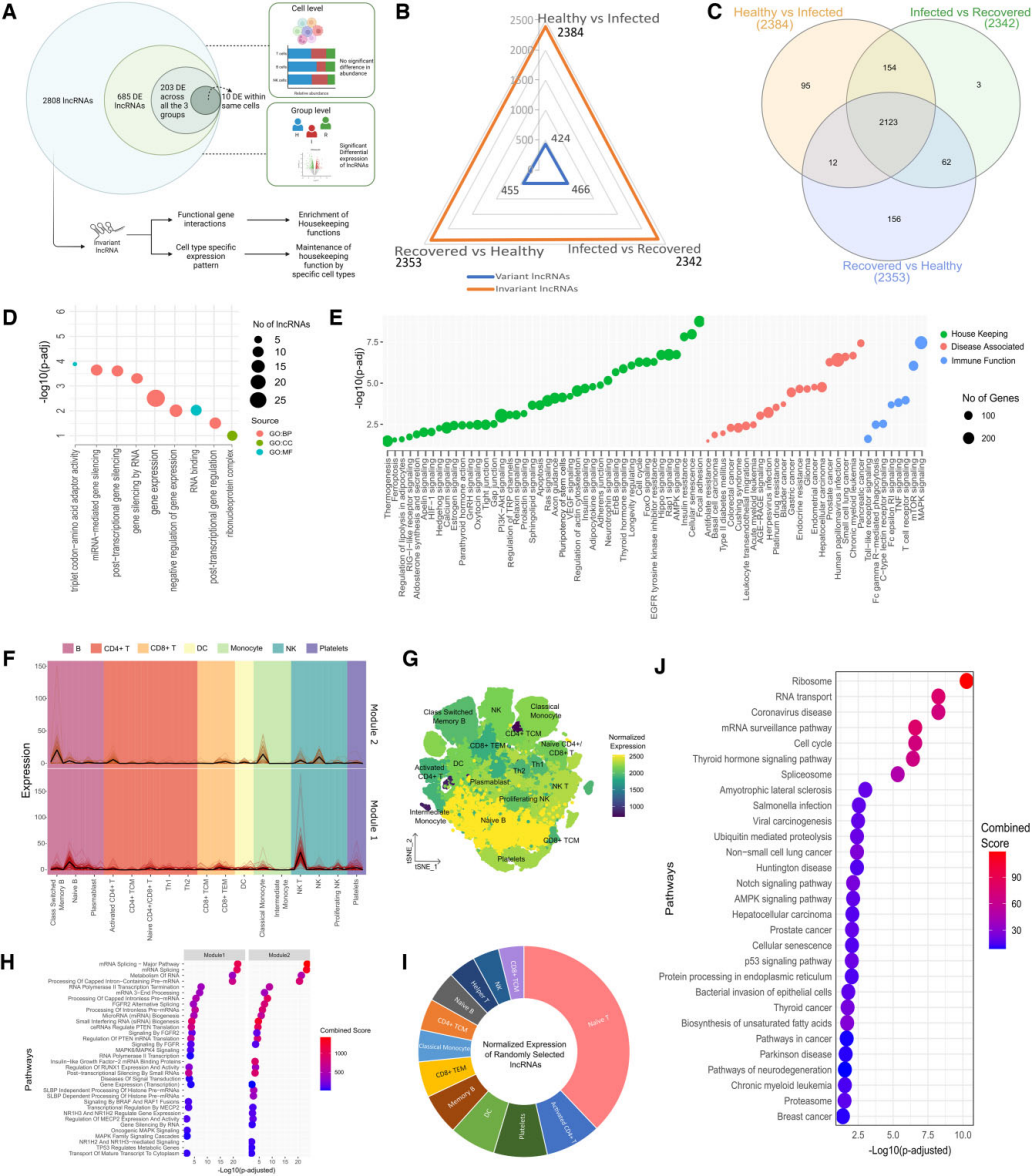
Cell type-specific expression pattern and functional role of invariant lncRNAs. (**A**) Graphical representation of the investigation of invariant lncRNAs. The study includes a greater proportion of invariant lncRNAs in addition to the DE lncRNAs segregated by cell type and group-specific enrichment. (**B**) Radar plot representing the number of variant and invariant lncRNAs between the three groups. The blue line represents the number of variant lncRNAs, and red indicates the invariant lncRNAs. (C) The Venn diagram represents the number of overlapping invariant lncRNAs between the three groups. (**D**) Annotation of invariant lncRNAs using the GO. The red dot indicates biological processes (BP), the green dot indicates cellular composition (CC), and the blue dot represents molecular functions (MF). (**E**) Gene set enrichment analysis of the invariant lncRNAs using experimentally validated interacting genes. (**F**) Cell type-specific functional expression modules of invariant lncRNAs. The cell subtypes have been grouped into major cell types and colored accordingly. (**G**) tSNE plot showing the expression profile of the invariant lncRNAs across all the cell types. The color represents the normalized expression (read counts) averaged with the number of cells for each cell type. (**H**) Pathway enrichment analysis of the lncRNAs falling under each module. (**I**) Average normalized expression of randomly selected 2123 lncRNAs across the cell types, as obtained from the EMBL Expression Atlas. (**J**) Pathway enrichment of the randomly selected lncRNAs using experimentally validated lncRNA-mRNA interaction information.

The DE lncRNAs and their cell type-specific expressions raise one question: despite having differential expression patterns across groups and specific cell types (Figure 1D, J), why was the overall lncRNA expression not different across the groups when looking at their cell type-specific expression (Figure 1E)? We propose that this may be due to a larger fraction of lncRNAs whose expression did not differ substantially across the healthy, infected, and recovered groups. As a result, we delved into exploring and elucidating the expressed yet invariant lncRNAs across the groups studied to learn more about their biological activities and cell-specific expression profiles (Figure 3A). Towards that, we found 2384 invariant lncRNAs between the infected and healthy, 2342 invariant lncRNAs between the infected and recovered, and 2353 invariant lncRNAs between the recovered and healthy. Importantly, we found overall 2123 lncRNAs to be invariant across all the conditions (Figure 3B,C). For example, expressions of *UCA1, SNHG18, SNHG27, SNHG28, ROR1-AS1, HOTAIR*, and *NRAV* did not change significantly from healthy to infected and recovered individuals. Notably, some of these lncRNAs are reported to be involved in infectious diseases. For example, *NRAV* (Negative Regulator of Antiviral Response) is reported to be involved in Influenza A virus (IAV) and respiratory syncytial virus (RSV) infection (2,48). *HOTAIR* is reported to be involved in cytokine production during hepatitis C virus (HCV) and Epstein-Barr virus (EBV) infection (49,50). Few of the lncRNAs in the SNHG family are known to be negative regulators of the inflammatory response, while *SNHG18*, *SNHG27*, and *SNHG28* are reported to be associated with different forms of cancer (51). Despite multiple reports of these lncRNAs being dysregulated during infection, we observed no significant difference in their expression across the healthy, infected, and recovered groups, and therefore, to understand their function, we performed Gene Ontology (GO) analysis of the invariant lncRNAs using gProfiler and observed enrichment of functions associated with gene silencing and post-transcriptional gene regulation, suggesting the invariant lncRNA-mediated gene regulation in the three groups (Figure 3D, Supplementary Table S5B). Based on the independently published experimentally validated interaction data, we also determined the mRNA regulated by these invariant lncRNAs to understand the biological roles of these lncRNAs (Supplementary Figure S2). We performed pathway enrichment analysis of the genes interacting with the invariant lncRNAs and, based on their biology, classified them into three functional groups. Interestingly, most of the pathways regulated by the invariant lncRNAs were found to be associated with housekeeping functions, namely signaling pathways, cell cycle regulation, and cell growth regulation (Figure 3E, Supplementary Table S5C).

To further understand the cell types that are benefiting from the invariant lncRNA-mediated regulation of housekeeping functions, we used CEMiTool to group the expression pattern of the invariant lncRNAs across the 17 cell types. Based on the expression pattern, two different expression modules were formed (module 1 and module 2), and class-switched memory B cells, naive B cells, classical monocytes, NK T cells, NK cells, and platelets were found to be the major contributors to the invariant lncRNA expression (Figure 3F). Surprisingly, none of the T cell populations was significantly associated with any of the expression modules. Therefore, we checked the expression profile of the invariant lncRNAs across all cell types and found significant reads of these invariant lncRNAs within the T cells (except CD4 + TCM) (Figure 3G). The pathway enrichment analysis of each of the two modules revealed significant enrichment of housekeeping functions, conforming to our previous result (Figure 3H, Supplementary Table S5D). This suggests that, in addition to their core immune tasks, class-switched memory B cells, naive B cells, classical monocytes, NK T, NK cells, and platelets, but not T cells, are engaged in sustaining housekeeping processes during and after SARS-CoV-2 infection.

Finally, we randomly selected an equal number of lncRNAs and checked their expression pattern and biological functions to see whether the enrichment of housekeeping functions is a common feature of lncRNAs. We have used the Blueprint Epigenome Project from the EMBL Expression Atlas to fetch the cell type-specific expression of the randomly selected lncRNAs and observed a distribution expression pattern across the cell types (Figure 3I). Pathway enrichment of the genes interacting with the random lncRNAs revealed several disease-associated pathways but not the housekeeping pathways as observed for our invariant lncRNAs, suggesting that the invariant lncRNA-mediated regulation of the housekeeping functions is a non-random event important for the host response during and after the SARS-CoV-2 infection (Figure 3J, Supplementary Table S5E).

### LncRNA-mRNA interaction and regulation modulate immune and inflammatory response

**Figure 4.**
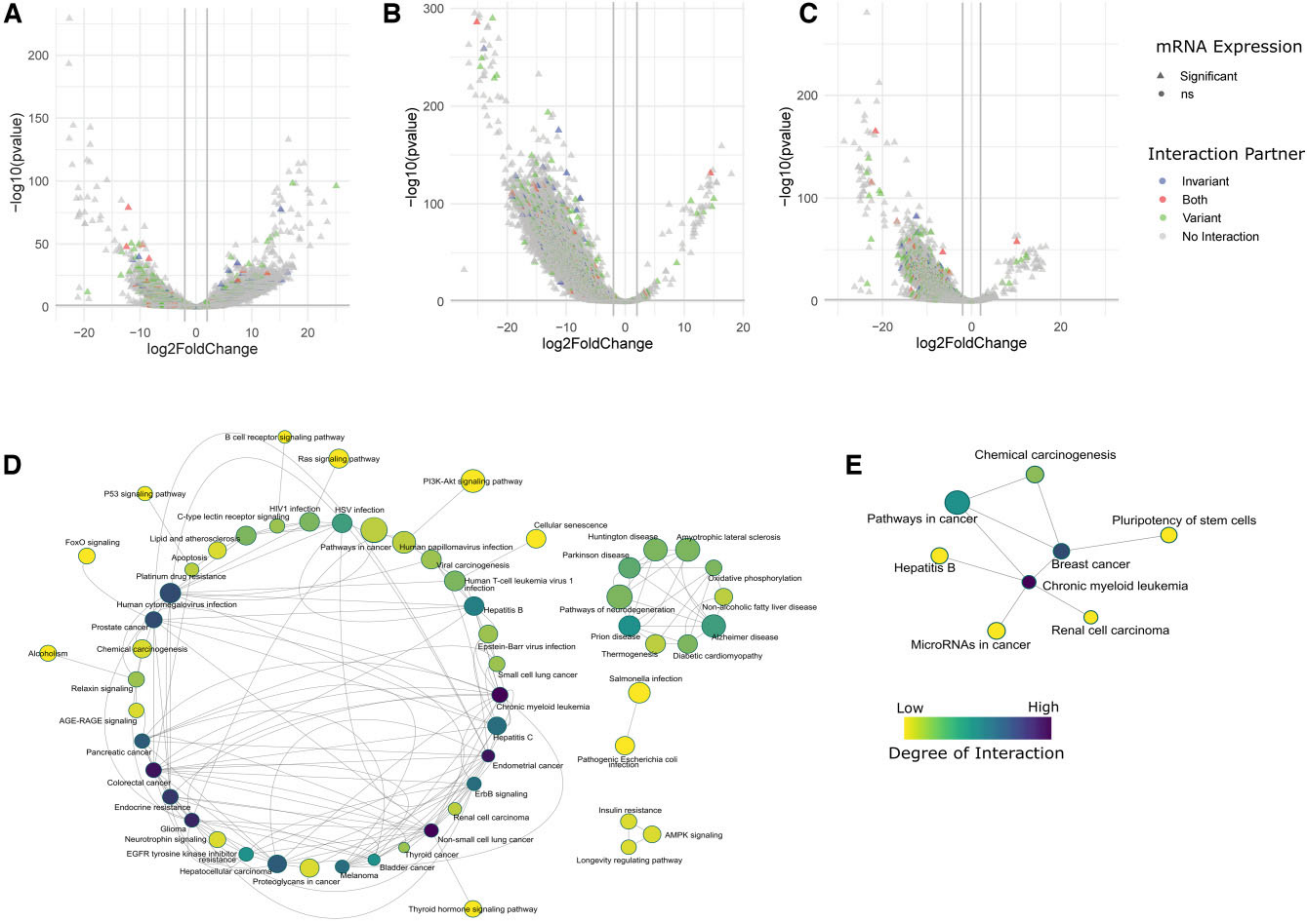
LncRNA-mRNA interaction and immune regulation. (A–C) Volcano plot showing differential expression of genes between (**A**) infected versus healthy, (**B**) recovered vs. infected, and (**C**) recovered vs. healthy. The significant DE genes have been presented in triangle shape, and the color denotes their interaction with lncRNAs. (D, E) Pathway enrichment of DE genes having interactions with (**D**) variant lncRNAs and (**E**) invariant lncRNAs. The network connections between pathways were established by using the same genes regulating multiple pathways, and the node size denotes the enrichment score of the specific pathway. The colour of the nodes represents the degree of interaction between the pathways, where a higher degree of interaction indicates multiple pathways being regulated by a similar group of genes.

Since the majority of the lncRNA functions are derived from the genes interacting with those lncRNAs, we wanted to see their expression in our data and whether they actually modulate those functions. We performed pseudo-bulk differential gene expression analysis across all three comparison groups. We observed 14 927 DE genes (Log_2_ fold change ≶ |2| and p adjusted value ≤ 0.05) in healthy vs. infected (10 676 upregulated, 4251 downregulated in infected), 20 895 genes in recovered vs. infected (183 upregulated and 20 712 downregulated in recovered), and 16 009 genes in recovered versus healthy (148 upregulated, 15 861 downregulated in recovered) (Figure 4A-C, Supplementary Table S6A-S6C). Notably, of the 903 genes interacting with invariant lncRNAs, 631 were significantly differentially expressed across healthy, infected, and recovered populations, while of the 3077 genes interacting with variant lncRNAs, 2448 were significantly differentially expressed. To understand the function of these interacting genes differentially expressed across healthy, infected, and recovered populations, we performed gene set enrichment analysis with the differentially expressed genes having interactions with the variant and invariant lncRNAs. The ranked gene list was used as input and the total expressed genes as background using ShinyGO v0.77. Interestingly, the genes interacting with variant lncRNAs were mainly associated with signaling pathways (ErbB, Ras, PI3K/AKT, p53, FoxO) and infections (for example, HIV-1, HSV, CMV, and hepatitis C), suggesting their role in the immune response during infection (Figure 4D, Supplementary Table S5F). On the other hand, genes interacting with invariant lncRNAs were mainly associated with regulating cell cycle, cell growth, and cell division processes within multiple cancer types (Figure 4E, Supplementary Table S5G), supporting our previous finding of invariant lncRNA-mediated regulation of housekeeping functions in SARS-CoV-2-infected and recovered individuals.

### Functional importance of genomic features underlying variant and invariant lncRNAs

**Figure 5.**
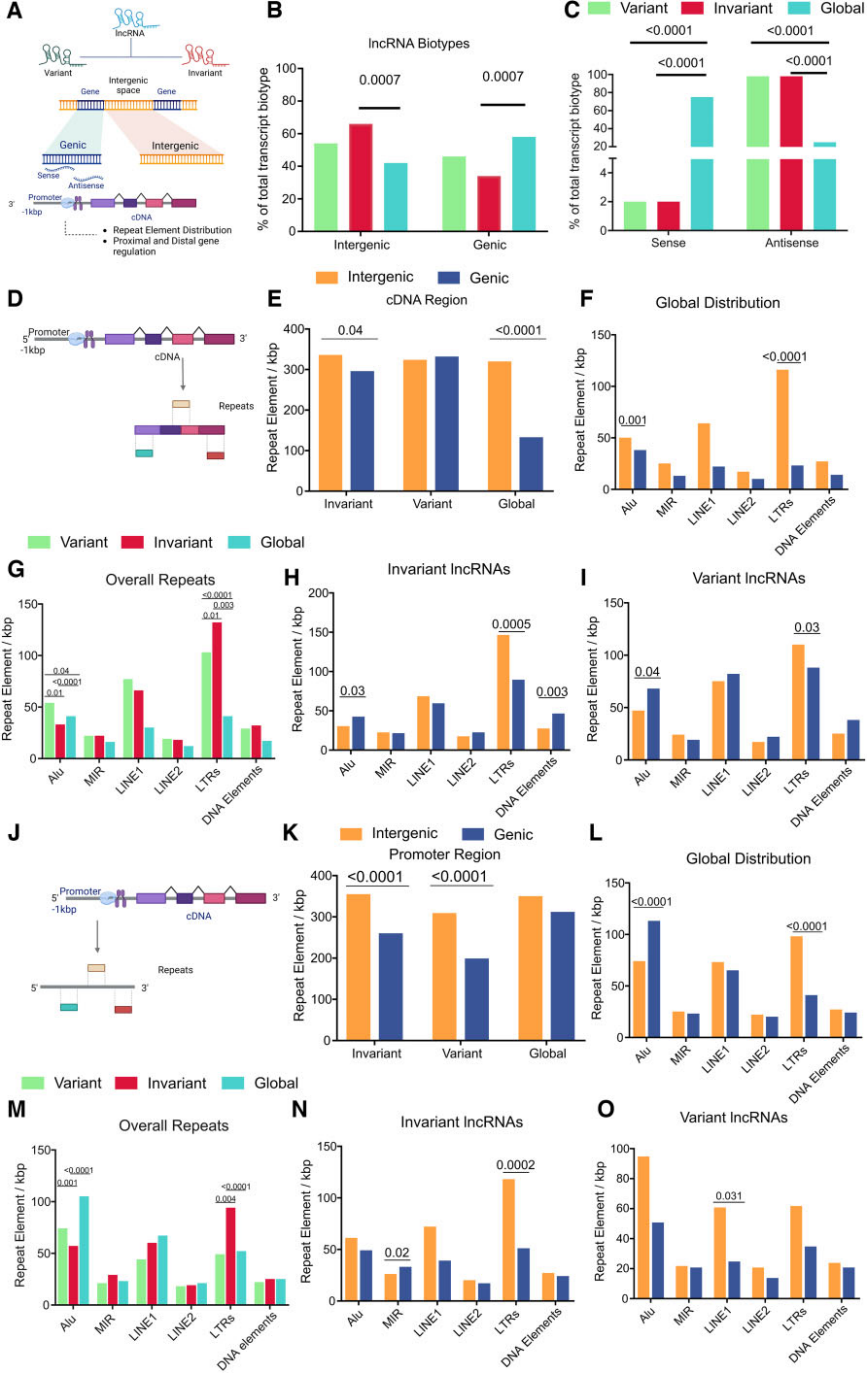
LncRNA biotype and repeat element distribution across the variant and invariant lncRNAs. (**A**) Graphical representation showing biotypes of variant and invariant lncRNAs and investigation of repeat element abundance within the cDNA and promoter regions of the lncRNAs. (**B**) Distribution of variant and invariant lncRNAs with respect to their origin from genic or intergenic origin, compared against global distribution. The data was represented as % of the total transcript biotype, and the colour of the bar represented the groups. The Chi-square test was applied for statistical significance. (**C**) Distribution of genic lncRNAs on sense and antisense between variant and invariant lncRNAs compared against global distribution of sense and antisense lncRNAs. (**D**) Graphical representation of querying repeat element abundance within the cDNA region of the lncRNAs. (**E**) Distribution of overall repeat bases per kbp of lncRNA length between variant and invariant lncRNAs as well as their origin. (**F**) Global distribution of repeat subfamilies within lncRNAs of genic and intergenic origin. (**G**) Overall distribution of repeat bases compared between the variant and invariant lncRNAs and their global abundance. (**H**) Distribution of repeat subtypes between the genic and intergenic invariant lncRNAs. (**I**) Distribution of repeat subtypes between the genic and intergenic variant lncRNAs. (**J**) Graphical representation depicting the abundance of repeats within the 1 kb upstream promoter region of the lncRNAs. (**K**) Overall distribution of repeat bases per kbp of lncRNA bases at the promoter region of variant and invariant lncRNAs. (**L**) Global distribution of repeat subtypes in the 1kb upstream promoter region. (**M**) Distribution of repeat subtypes compared between the promoter regions of variant and invariant lncRNAs. (N, O) Distribution of repeat subtypes within the promoter region between genic and intergenic, (**N**) invariant lncRNAs, and (**O**) variant lncRNAs. In figures E–I, K–O, repeat element abundance was expressed as repeat element bases per kbp of lncRNA length. In figures B–C, G, and M, the color of the bar represents the group of lncRNAs, where green represents variant lncRNA, red represents invariant lncRNA, and blue represents global lncRNA. In figures E-F, H-I, K-L, and N-O, the color of the bar represents the origin of the lncRNAs, where yellow represents lncRNAs of intergenic origin and blue represents lncRNAs of genic origin. A chi-square test was applied for the estimation of statistical significance between the comparison groups.

Given that the majority of lncRNAs remain invariant and exhibit distinct biological functions compared to variant lncRNAs, it becomes crucial to explore the genomic features and regulatory mechanisms that differentiate these two categories. Repeat elements constitute approximately 45% of the human genome and are key components in regulatory mechanisms (52). They are also reported to be associated with and modulate infectious disease severity (5,25,26). Therefore, we segregated the lncRNAs based on their biotype and origin (using LncRBase v2.0) and then investigated the repeat element distribution within the cDNA and promoter regions of the lncRNAs (Figure 5A). We observed a significant proportion of the invariant lncRNAs to be of intergenic origin compared to the global distribution of genic and intergenic lncRNAs (Figure 5B, Supplementary Table S7). We further classified the lncRNAs of genic origin with respect to the strand they are present in and observed that 98% of both variant and invariant lncRNAs were present in the antisense strand, which was significantly different from the global distribution of sense and antisense lncRNAs (Figure 5C).

We then looked at the repeat element distribution within the body of both the variant and invariant lncRNAs (Figure 5D, Supplementary Table S8). We observed a significantly higher distribution of repeats in the invariant lncRNAs of intergenic origin compared to the genic invariant lncRNAs. This followed the global trend of a higher abundance of repeats in the intergenic lncRNAs (Figure 5E). It is important to note that globally, Alu and long terminal repeat (LTR) elements are more abundant in the intergenic lncRNAs compared to the genic lncRNAs (Figure 5F). Interestingly, even though the abundance of repeats was not significant within the variant lncRNAs, we found a significantly higher abundance of Alu and LTR elements in the variant lncRNAs when compared to their global distribution (Figure 5G). For the invariant lncRNAs, the abundance of Alu repeats was lower than both the variant and global lncRNAs, while the LTRs were more abundant than both variant and global lncRNAs. Contrary to the global pattern, the abundance of Alu elements was higher in both the invariant and variant genic lncRNAs (Figure 5H, I). LTR elements were more abundant in both the variant and invariant intergenic lncRNAs, similar to the global distribution of LTRs between the genic and intergenic lncRNAs. Overall, this evidence implies that while variant and invariant lncRNAs have similar repeat element abundances, they diverge significantly from the global pattern of repeat elements.

Since the promoter region is the key regulatory region of both coding and noncoding genes, we investigated the abundance of repeats within the promoter region to understand the possible differences in repeat-mediated regulation of lncRNA expression between the variant and invariant lncRNAs. We selected a region of 1 kb upstream to the transcription start site (TSS) and analyzed for repeats (Figure 5J). We observed a significantly higher abundance of repeats in the promoter region of intergenic lncRNAs of both the variant and invariant lncRNAs compared to the genic variant and invariant lncRNAs. While this followed the global trend of repeat element abundance, the difference between the global repeats and variant/invariant repeats was not significant at the promoter region (Figure 5K, Supplementary Table S8B). Unlike the cDNA region, the global trend shows a higher abundance of Alu in the promoter region of genic lncRNAs, while the LTR abundance was higher in the promoter region of intergenic lncRNAs (Figure 5L). Interestingly, we observed a lower abundance of Alu in the promoter region of both the variant and invariant lncRNAs compared to the global abundance of Alu. However, the distribution between genic and intergenic lncRNAs was not significant statistically, contrary to their global abundance (Figure 5M–O). The abundance of LTR elements was significantly high in the promoter region of invariant lncRNAs compared to both the variant and global distribution (Figure 5M). Mammalian-wide interspersed repeat (MIR) and LTRs were also significantly different between the genic and intergenic invariant lncRNAs, while only long interspersed nuclear element 1 (LINE1) abundances were significantly higher at the promoter region of the intergenic variant lncRNAs. Overall, the findings suggest that both the variant and invariant lncRNAs are similar in terms of the repeat element abundance pattern at both the cDNA and promoter regions. This itself is a significant finding considering the deviation from the global abundance of repeats and the different expression dynamics between variant and invariant lncRNAs despite a similar abundance of repeats. Therefore, the findings make a case for further investigation of invariant lncRNA function during infections.

### Increased proximal and distal gene interaction by the variant lncRNAs

**Figure 6.**
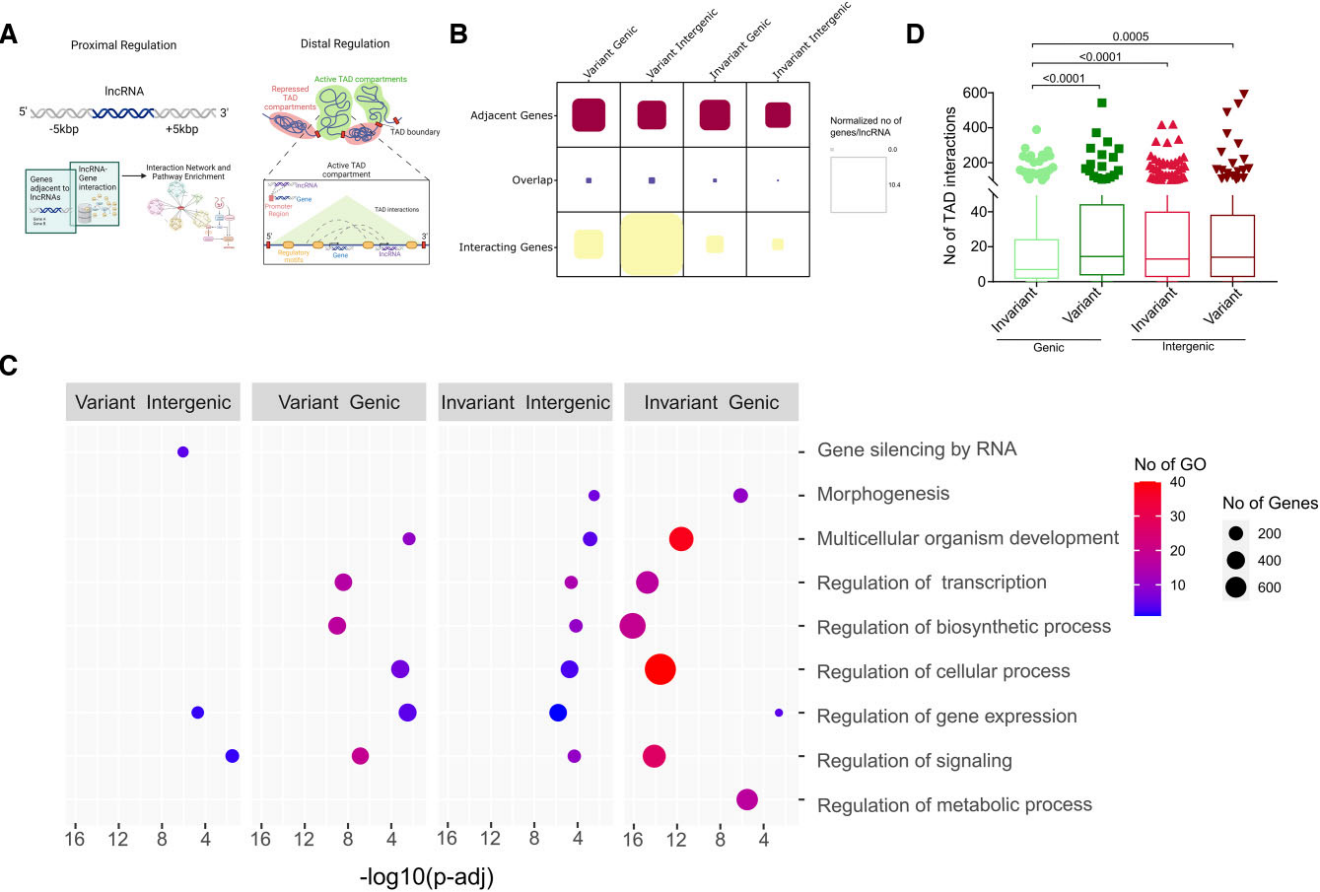
Proximal and distal gene interaction by the variant and invariant lncRNAs. (**A**) Graphical representation depicting investigation of proximal and distal gene interactions. (**B**) Average number of adjacent genes, known interacting genes, and interacting genes present within the ±5 kb region of the lncRNAs. (**C**) Pathway enrichment analysis of the lncRNAs having interactions with the proximal genes. (**D**) Number of interactions between lncRNAs and promoter regions of protein-coding genes present within the same TAD.

Since we observed that the abundance of repeats at both the cDNA and promoter regions is similar between the variant and invariant lncRNAs, and based on our earlier findings of different degrees of interactions between the protein-coding gene and the cell-type-specific and nonspecific DE lncRNAs, we investigated the gene regulation and interaction with the protein-coding genes (present in proximal and distal locations) by both the variant and invariant lncRNAs. For proximal regions, we selected a region of 5 kb upstream and downstream of the lncRNA and identified the genes present within the flanking region. We used the retrieved interacting mRNAs from the lncRNA-mRNA network across the two datasets (variant and invariant lncRNAs) and identified the genes that are in close vicinity to the lncRNAs and have known interactions with the protein-coding genes (Figure 6A). Overall, we observed a similar number of proximal gene interactions per lncRNA, irrespective of their origin (genic and intergenic) and type (variant and invariant). However, interestingly, the variant lncRNAs had a significantly higher number of interactions (p value < 0.0001) with the proximal genes than the invariant lncRNAs (Figure 6B, Supplementary Table S9A). This is possibly because the variant lncRNAs are primarily involved in the regulation of immune responses, which require more interaction partners compared to the invariant lncRNAs, which are mainly involved in housekeeping functions. To test this hypothesis, we performed pathway enrichment analysis with the lncRNAs having interactions with proximal genes and observed that the contribution to the housekeeping functions by the variant lncRNAs with proximal gene interactions is much less than the invariant lncRNAs with proximal gene interactions (Figure 6C, Supplementary Table S5H). Further, we have checked the lncRNA interactions with distally located mRNAs as well (Figure 6A). For this, we have used the Topologically Associating Domain Knowledge Base (TADKB) database to fetch the Topologically Associated Domains (TAD) boundaries detected by Hi-C in K562 cells at a 10 kb resolution (Supplementary Table S9A). We then identified the TADs corresponding to the lncRNAs and other protein-coding genes from the same TAD. We used the Triplex Domain Finder to identify any interactions between the promoter region of protein-coding genes and the lncRNA present within the same TAD.

We observed that variant lncRNAs have a significantly higher number of interactions with the protein-coding genes present within the same TAD compared to the invariant lncRNAs (Figure 6D, Supplementary Table S9B). This lends support to our previous finding of higher interactions between the variant lncRNAs and proximal genes. Notably, we earlier observed a higher abundance of Alu in the variant lncRNAs compared to the invariant (Figure 5G). In our previous studies, we have shown Alu-mediated regulation of proximal genes as well as homologous interaction with the promoter region of protein-coding genes present within the same TAD (53–55). Therefore, the findings suggest that Alu-mediated interactions between proximal as well as distal genes and variant lncRNAs are more likely to facilitate immune regulation in SARS-CoV-2-infected and recovered individuals.

## Discussion

Despite numerous findings highlighting the role of lncRNAs in infectious diseases and the flourishing scope and applications of single-cell RNA-seq, there have been very few studies on the single-cell resolved lncRNA response in different disease contexts. For example, Liu et. al. investigated the role of lncRNAs in developing human neocortex; Shaath et. al. investigated the role of lncRNAs in triple-negative breast cancer; Santus et. al. investigated the role of lncRNAs in Ebola virus infection; Huang *et al.* and Aznaourova *et al.* in COVID-19 (28,56–59). In this study, we aim to understand the functional role of the poly-adenylated mature lncRNAs in the SARS-CoV-2-infected and recovered individuals at single cell resolution. We performed differential expression analysis of the lncRNAs and identified 203 lncRNAs that were upregulated in the infected compared to the healthy but downregulated in the recovered compared to both the infected and healthy. While a small fraction of the DE lncRNAs (10 lncRNAs, *MALAT1, SNHG25, NEAT1, SNHG7, LINC01215, ILF3-DT, EBLN3P, ZFAS1, EPB41L4A-AS1*, and *SNHG8*) were differentially expressed within the same cell type across healthy, infected, and recovered groups, the other DE lncRNAs (193 lncRNAs, for example, *BISPR, SNHG9, NORAD, NIFK-AS1, CYTOR*) were differentially expressed in the different cell types across the three groups. The lncRNA-mRNA interaction network (based on an interaction database) and pathway enrichment analysis revealed regulation of pathways like TGF beta, mTORC1, and PI3K/AKT/mTOR signaling exclusively by the cell-type-specific DE lncRNAs. These pathways are primarily involved in the immune response cascade during an infection. *MALAT1* and *NEAT1* have been experimentally validated to modulate immune responses during different infections (28,60,61). Aznaourova *et al.* validated *LUCAT1* as a regulator of systemic monocyte immunity during COVID-19 (59). Another lncRNA, *NORAD*, has been established to be associated with MTb infection (62). The nonspecific DE lncRNAs, on the other hand, are involved in mainly DNA repair, cell growth, and cell division pathways. The differential gene expression analysis and lncRNA interaction with DE genes, followed by pathway enrichment analysis from our data, also support the above finding. For example, *MALAT1* interacts with *MYC, MAPK1, LTBP3*, and *TNF*, all of which were upregulated in the infected and downregulated in the recovered and are associated with TGF beta signaling, a key pro-inflammatory signaling pathway. Both the interaction database and our data show a higher number of genes interacting with variant lncRNAs than the invariant lncRNA. This suggests that the cell type-specific DE lncRNAs require a higher degree of interaction with the mRNAs to modulate the immune response pathways within NK T, proliferating NK, plasmablast, classical monocytes, dendritic cells, naive B, naive T, activated CD4 + T, and class-switched memory B cells during and after SARS-CoV-2 infection.

While it is important to understand the differential host response to infection, it is also important to identify the component of the host response that did not change upon a pathogen challenge and understand the potential regulation of the invariant host response and its functional role. We identified 2123 lncRNAs (for example, *UCA1, SNHG18, SNHG27, SNHG28, ROR1-AS1, HOTAIR*, and *NRAV*) with no significant change in expression across healthy, infected, and recovered individuals. Based on the lncRNA-mRNA interaction (both the interaction database and our data), we observed a regulation of housekeeping functions by the invariant lncRNAs across healthy, infected, and recovered individuals. The majority of these housekeeping functions were signaling pathways associated with cell growth, division, senescence, and transcriptional regulation. For example, focal adhesion, cellular senescence, AMPK, and Rap1 signaling are top-enriched pathways involved in cellular metabolism, growth regulation, membrane contraction, and cytoskeletal changes. Some of the invariant lncRNAs have also been reported in other disease contexts as well. For example, *ROR1-AS1* has been reported to promote cell growth and regulation of gene transcription in bladder cancer and mantle cell lymphoma (63,64). *SNHG18* was reported to enhance cell motility and cytoskeletal remodeling in glioma cells (51). *UCA1* facilitates cell proliferation and inhibits apoptosis in retinoblastoma by activating the PI3K/Akt pathway (65). Another lncRNA, *HOTAIR*, was reported to be involved in protein ubiquitination (66). While modulation of these pathways is crucial in the pathophysiology of specific diseases, they are also important housekeeping functions in other disease contexts. These pathways are also critical during SARS-CoV-2 infection since the virus hijacks the cellular machinery to replicate within the host and, as a consequence, alters these housekeeping functions (67–69). One classic example is *HOTAIR* interaction with *HNRNPM, FUS, HNRNPA1*, and *HNRNPU*, all of which were downregulated in the recovered and are associated with spliceosome-mediated transcriptional regulation of gene expression. It is worth noting that spliceosome-mediated transcriptional regulation is one of the dysregulated processes in SARS-CoV-2-infected individuals. The cell type-specific expression modules highlighted NK T, classical monocytes, NK cells, naive B, class switched memory B, activated CD4+ T cells, and platelets as major contributors to the invariant lncRNA expression. Interestingly, the NK T, classical monocytes, naive B, and class switched memory B cells were also associated with variant lncRNA-mediated immune response regulation, suggesting dual functions of these cells during COVID-19 as well as in recovered patients.

While investigating the regulation of invariant lncRNA expression, we observed that, unlike the global pattern, more invariant lncRNAs were of intergenic origin. The intergenic space in the human genome is mainly occupied by noncoding genes and transposable elements (5–7,70,71). Notably, the abundance of Alu elements was higher in the variant lncRNA, especially those of genic origin, contrary to the higher global abundance of Alu in the intergenic lncRNAs. Various studies, including our previous work, have highlighted Alu-mediated interactions between the lncRNAs and mRNAs, supporting the observed higher degree of interaction between the variant lncRNAs and proximal/distal genes (5–7,25,26). Alluding to our previous findings (*MALAT1* interaction with *MYC, MAPK1, LTBP3*, and *TNF*), *LTBP3* is present within the same TAD as in *MALAT1*, and the abundance of repeats as well as interaction analysis between *LTBP3* and *MALAT1* suggest a possible Alu-mediated homologous interaction between the promoter region of *LTBP3* and *MALAT1*, supporting our hypothesis of higher repeat-mediated interactions between the variant lncRNA and proximal/distal genes. Our analysis also suggested a possible interaction between *HOTAIR* and *HNRNPA1*, suggesting shared regulatory mechanisms for both the variant and invariant lncRNAs. Besides, Alu elements are known to harbor various transcription factor binding sites and therefore modulate the expression of the genes and lncRNAs (5–7,25,26,72,73). The higher abundance of Alu on the variant lncRNAs also suggests that the variant lncRNAs are subject to more dynamic regulation compared to the invariant lncRNAs during and after COVID-19. Notably, we observed a high abundance of LTR repeats within both variant and invariant intergenic lncRNAs. While few studies reported the LTRs acting as promoters for viral gene transcription and facilitating viral replication, a recent study reported that the LTR repeats are likely to bind to the ribosome and therefore possibly modulate the translation process, which warrants further research to unfold the function of LTRs (5–7,74,75). Overall, the similar dynamic abundance of repeat elements within variant and invariant lncRNAs suggests a similar yet non-random repeat-mediated regulation of both variant and invariant lncRNA expression.

In summary, we have identified two distinct patterns of lncRNA expression: variant and invariant, across healthy, SARS-CoV-2-infected, and recovered individuals. While the variant lncRNAs are primarily involved in the regulation of immune responses, the invariant lncRNAs are responsible for maintaining critical housekeeping functions. Notably, we observed NK T, naive B, class switched memory B, and classical monocytes to participate in lncRNA-mediated maintenance of housekeeping functions in addition to their primary immune functions. The similar non-random, dynamic distribution of repeat elements within variant and invariant lncRNAs suggests similar regulation of expression for both variant and invariant lncRNAs, making the invariant lncRNAs an important candidate for further research to understand the maintenance of homeostasis during and after SARS-CoV-2 infection.

The study offers a thorough insight into the cell type-specific functions of lncRNAs during and after COVID-19 at a single-cell resolution. However, it is important to note that the findings are reliant on polyadenylated lncRNAs, given the current limitations of available single-cell capturing methods. Exploring the entire non-coding transcriptome, including non-polyadenylated RNAs, might unveil additional cell-type-specific functions. At the same time, findings would be strengthened by studies in other study cohorts, both within India and globally. Insights from other infectious diseases, particularly those caused by RNA viruses, would be required to carry forward the findings presented in this manuscript. Especially, longitudinal studies after recovery from the disease would be important to validate the immune dynamics highlighted in the recovered COVID-19 patients in this study. Another limitation of the present study is the unfeasibility of validating identified genes via qPCR due to challenges in obtaining a sufficient quantity of clinical samples during the early phase of the COVID-19 pandemic and the inability to sub-sample RNA from lysed cells during downstream single-cell library preparation.

## Supplementary Material

lqae023_Supplemental_Files

## Data Availability

All raw and processed sequencing data generated in this study have been submitted to the NCBI Gene Expression Omnibus (GEO; https://www.ncbi.nlm.nih.gov/geo/) under accession number GSE201088.
