# Supplementary material for: Cell-specific housekeeping role of lncRNAs in COVID-19-infected and recovered patients
Source: NAR Genom Bioinform. 2024 Feb 29;6(1):lqae023. doi: 10.1093/nargab/lqae023 (PMC10903533; doi:10.1093/nargab/lqae023)
Supplement: lqae023_Supplemental_Files [file lqae023_supplemental_files.zip › Supplementary Figures.docx]

**
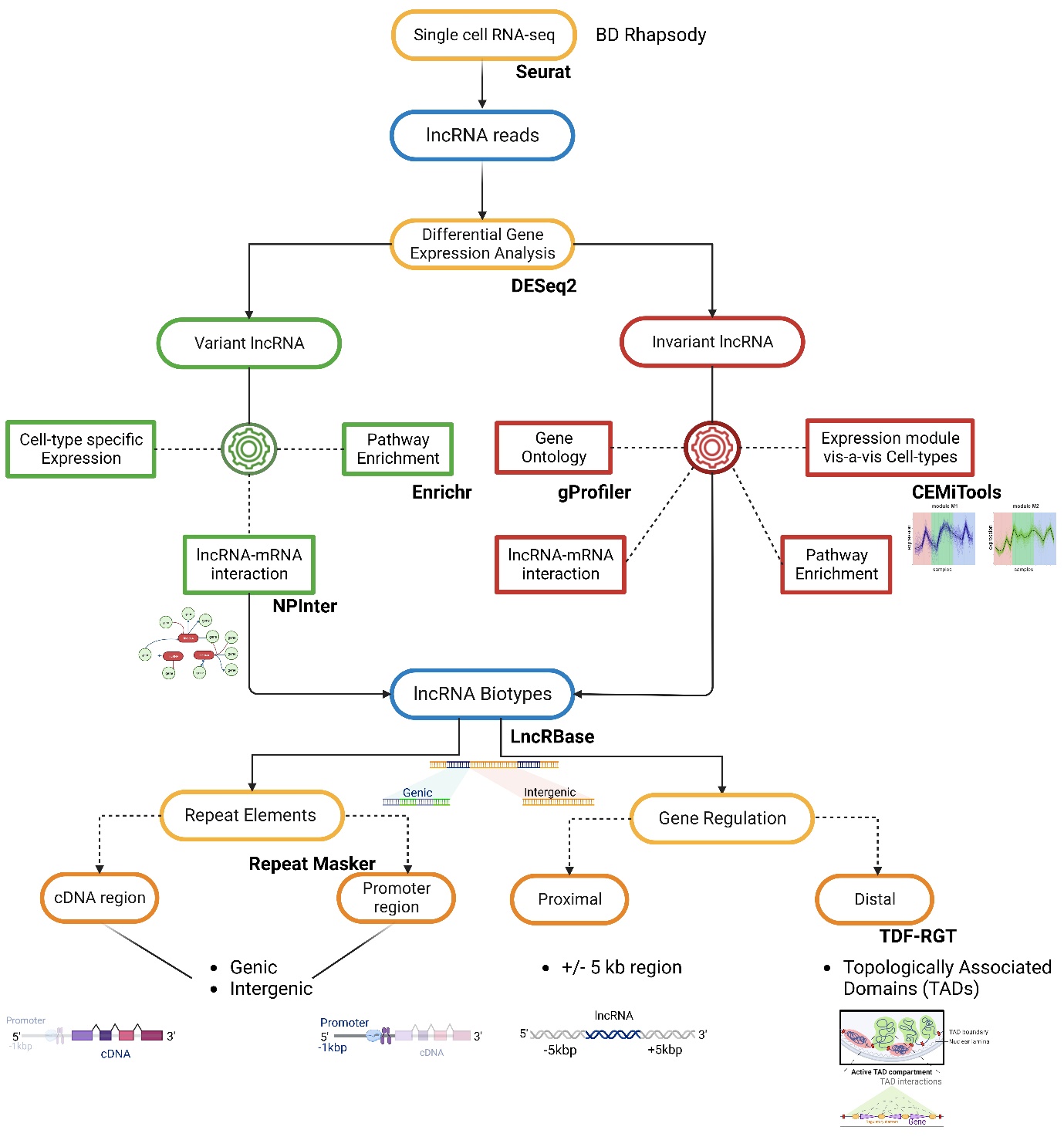
**

**Supplementary Figure 1:** **Experimental workflow for identification and functional understanding of the lncRNAs from the healthy, infected and recovered individuals at single cell resolution.** lncRNA reads were collected from the single cell dataset and subjected to differential expression analysis, yielding variant and invariant lncRNAs. Pathway enrichment and lncRNA-mRNA interactions were investigated for both sets of lncRNAs. Cell type specific expression was examined for variant lncRNAs, whilst CEMiTools was used to create invariant expression modules vis-à-vis cell types. All of the lncRNAs were then classified based on their biotypes and repeat distribution - at the cDNA and promoter regions - and their proximal and distal interactions were investigated.


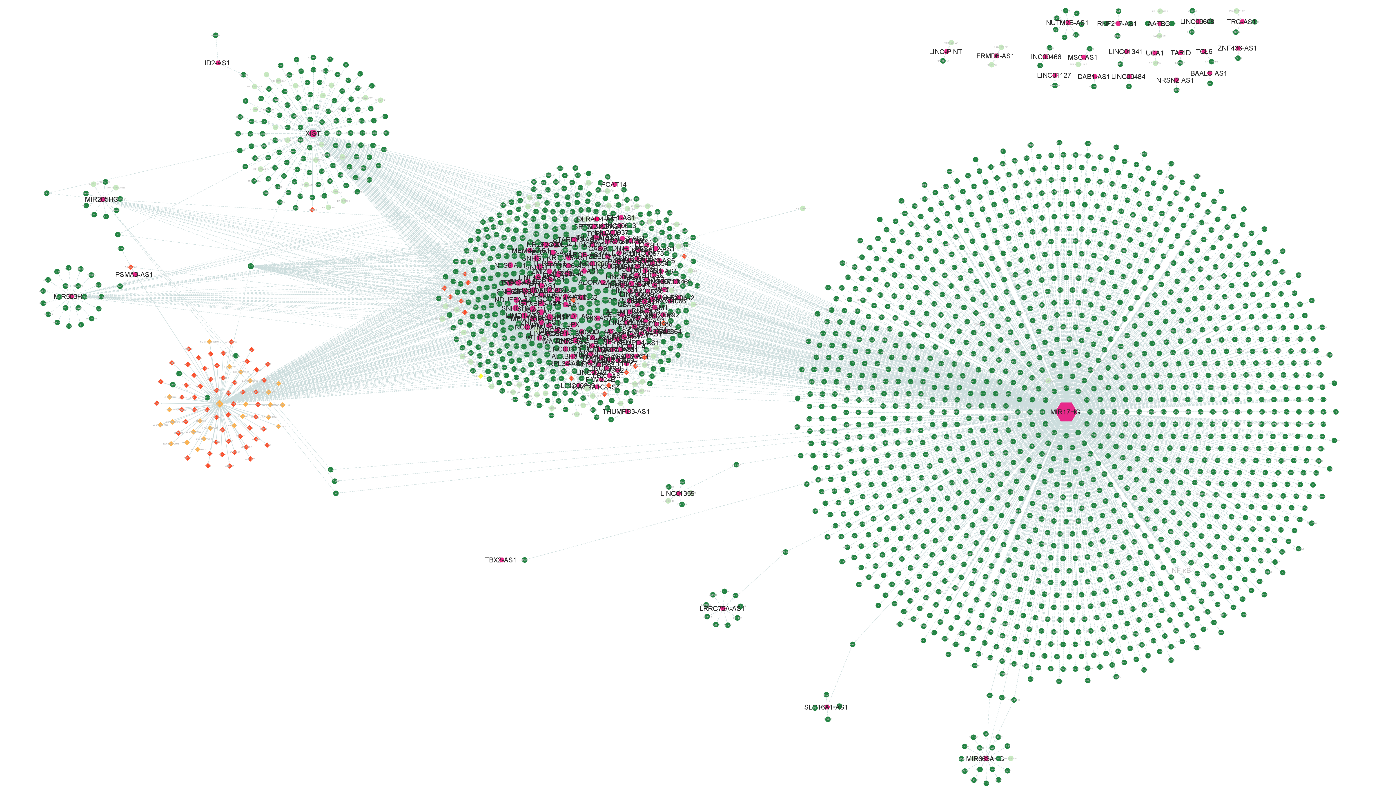


**Supplementary Figure 2:** **LncRNA-mRNA interaction network of invariant lncRNAs.** The network represents lncRNA-mRNA interaction for invariant lncRNAs. Green nodes represent interacting mRNAs (dark green for regulatory interactions; light green for binding interactions), pink nodes represent invariant lncRNAs, orange nodes represent interacting lncRNA partners (light orange for regulatory interactions; dark orange for binding interactions). Diamond node shapes represent regulatory interaction partners, whereas circles represent binding interaction partners.

**List of Supplementary Files**

Supplementary Figure 1: Experimental workflow

Supplementary Figure 2: LncRNA-mRNA interaction network of invariant lncRNAs

Supplementary Table S1: Patient Demographics and Clinical details

Supplementary Table S2A: DE analysis of lncRNA between Healthy vs Infected

Supplementary Table S2B: DE analysis of lncRNA between Recovered vs Infected

Supplementary Table S2C: DE analysis of lncRNA between Recovered vs Healthy

Supplementary Table S3A: Specific, nonspecific de lncRNAs

Supplementary Table S3B: Functions of the variant and invariant lncRNAs in other diseases

Supplementary Table S4A: Variant lncRNA interaction

Supplementary Table S4B: Invariant lncRNA interaction

Supplementary Table S5A: Pathway enrichment for specific and non-specific DE

Supplementary Table S5B: GO of invariant lncRNA

Supplementary Table S5C: Pathway of invariant lncRNA

Supplementary Table S5D: Pathway of modules

Supplementary Table S5E: Pathway of Random lncRNA

Supplementary Table S5F: Pathway of variant interacting genes

Supplementary Table S5G: Pathway of invariant interacting genes

Supplementary Table S5H: Pathway of proximal genes

Supplementary Table S6A: DE genes between Infected vs Healthy

Supplementary Table S6B: DE genes between Infected vs Recovered

Supplementary Table S6C: DE genes between Healthy vs Recovered

Supplementary Table S7: Transcript Biotype analysis

Supplementary Table S8A: Repeat abundance within lncRNA body

Supplementary Table S8B: Repeat abundance within promoter region of lncRNA

Supplementary Table S9A: Proximal and Distal gene list

Supplementary Table S9B: TAD Interaction
